# Status of Lymph Node Metastasis and Efficacy of Lymph Node Dissection Along the Superior Mesenteric Artery in Pancreatic Head Cancer: Clinicopathological Analysis of Patients Undergoing Pancreatoduodenectomy With Circumferential Lymph Nodes Dissection Along the Superior Mesenteric Artery

**DOI:** 10.1002/jhbp.70071

**Published:** 2026-01-28

**Authors:** Isamu Makino, Hirohisa Kitagawa, Ryohei Takei, Kaichiro Kato, Satoshi Takada, Mitsuyoshi Okazaki, Yasuhito Iwao, Shinichi Nakanuma, Tetsuo Ohta, Shintaro Yagi

**Affiliations:** ^1^ Department of Hepato‐Biliary‐Pancreatic Surgery and Transplantation Kanazawa University Kanazawa Ishikawa Japan

**Keywords:** lymph node dissection, lymph node metastasis, pancreatic head cancer, superior mesenteric artery

## Abstract

**Background:**

Between 2002 and 2013, we performed pancreatoduodenectomy (PD) with combined resection of the superior mesenteric artery (SMA) (PD‐SMAR) to treat locally advanced pancreatic head cancer (PHC). Since 2011, we have performed PD with circumferential lymph node dissection along the SMA (PD‐CLDS), in which we dissected lymph nodes all around the SMA for thorough lymph node dissection (LND) along the SMA. In this study, we examined the status of lymph node metastasis and the efficacy of LND along the SMA.

**Methods:**

In the first study, 22 patients with PHC who underwent PD‐SMAR were enrolled. We examined the location of lymph nodes along the SMA and the incidence of metastasis. In the second study, we examined the incidence of metastasis at each lymph node station and evaluated the efficacy of LND along the SMA with 103 patients who underwent PD‐CLDS.

**Results:**

Lymph nodes along the SMA were classified into five basins using PD‐SMAR specimens. Lymph nodes along the SMA were the most frequent station of metastasis, which showed the highest efficacy of LND in PD‐CLDS.

**Conclusions:**

Lymph nodes along the SMA are a frequent site of metastasis and LND along the SMA demonstrates high efficacy among the regional lymph nodes in PHC.

## Graphical abstract

Makino and colleagues analyzed 103 patients undergoing pancreatoduodenectomy with circumferential lymph node dissection along the superior mesenteric artery for pancreatic head cancer. Lymph nodes along the superior mesenteric artery were the most frequent site of regional metastasis and showed the highest efficacy index of dissection among regional lymph node stations.

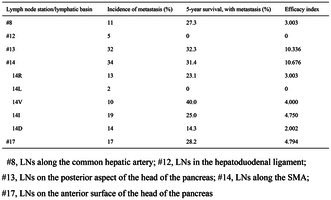

## 1 Introduction

Lymph node (LN) metastasis is one of the important prognostic factors in pancreatic cancer [1, 2, 3, 4]. Because pancreatic head cancer (PHC) often extends toward the superior mesenteric artery (SMA) [5, 6, 7], recognizing the lymphatic pathway and accurate location of the LNs along the SMA are essential to adequately evaluate the metastatic status and the impact of lymph node dissection (LND) along the SMA. Due to the anatomical complexity and difficulty of dissection along the SMA during pancreatic resection, the optimal extent and dissecting procedure for LNs along the SMA have not been established [8, 9, 10, 11]. Consequently, the prognostic significance of LND along the SMA remains unclear.

Between 2002 and 2013, we performed pancreatoduodenectomy (PD) with combined resection of the SMA (PD‐SMAR) for locally advanced PHC [5, 6]. We obtained en‐bloc surgical specimens of the pancreatic head and the SMA during this procedure. The precise location of the LNs along the SMA could be recognized by pathological examination of those en‐bloc surgical specimens.

In this study, we first aimed to investigate the location of LNs along the SMA and classify the nodes based on their anatomical relationship with the SMA. Then, we evaluated the incidence of metastasis to each LN in patients who underwent PD‐SMAR.

Since our first study showed frequent metastasis to LNs all around the SMA in the PD‐SMAR specimens, we started routinely dissecting LNs along the SMA circumferentially for patients with PHC.

In the second study, we evaluated the efficacy of LND along the SMA for PHC using clinicopathological analysis of the patients who underwent pancreatoduodenectomy with circumferential LND along the SMA (PD‐CLDS).

## 2 Methods

### 2.1 Study 1: Pathological Study of LNs Along the SMA in PD‐SMAR

#### 2.1.1 Patients

Between 2002 and 2013, 66 patients with PHC underwent PD at Kanazawa University Hospital. During this period, we performed PD‐SMAR for patients under 80 years of age with good performance status when PHC progressed toward the SMA and R0 resection was expected through concomitant resection of the SMA. Twenty‐two of the 66 patients who underwent PD‐SMAR for PHC were enrolled in the first study.

#### 2.1.2 Pathological Examination

**FIGURE 1:**
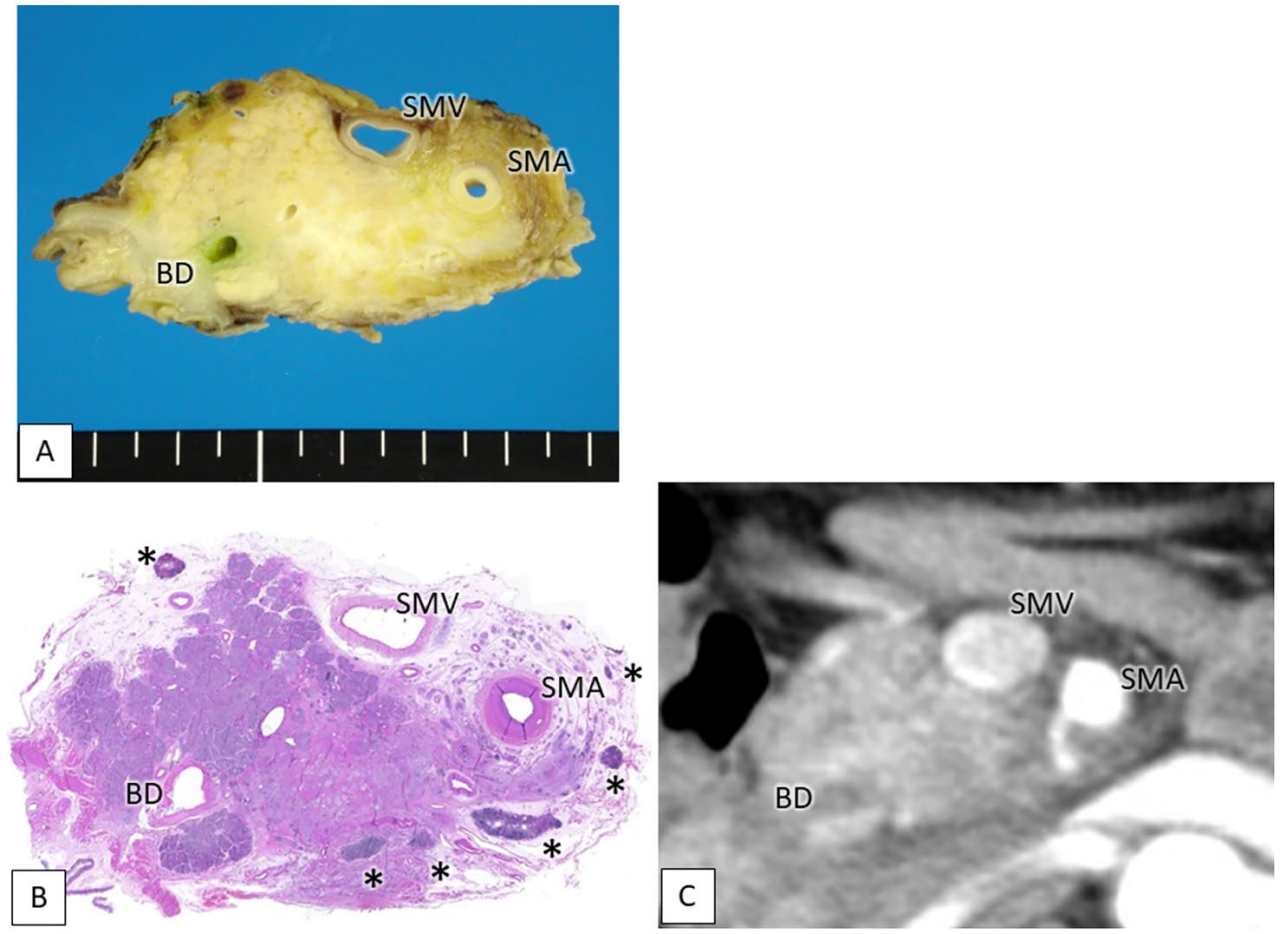
Pathological examination in study 1. (A) The resected specimen was cut horizontally into 5‐mm tissue blocks to correspond to the preoperative CT axial image. (B) We observed the location of lymph nodes along the SMA and evaluated the incidence of metastasis to each node (H&E, original magnification ×1). (C) We observed the specimen in contrast with CT images of the corresponding slices during pathological analysis. Asterisk (*) indicates lymph nodes. BD, bile duct; SMA, superior mesenteric artery; SMV, superior mesenteric vein.

The resected specimen was cut horizontally into 5‐mm tissue blocks to correspond to the preoperative computed tomography (CT) axial image. Histopathological examinations were performed using standard hematoxylin and eosin (H&E) staining. We identified and mapped the LNs along the SMA and classified them according to their spatial relationship with the SMA. During pathological analysis, we observed the specimen in contrast to the CT axial images of the corresponding slices to recognize the precise anatomical orientation (Figure 1). Then, we evaluated the incidence of metastasis to each node.

### 2.2 Study 2: Evaluation of Efficacy of LND Along the SMA

#### 2.2.1 Patients

**FIGURE 2:**
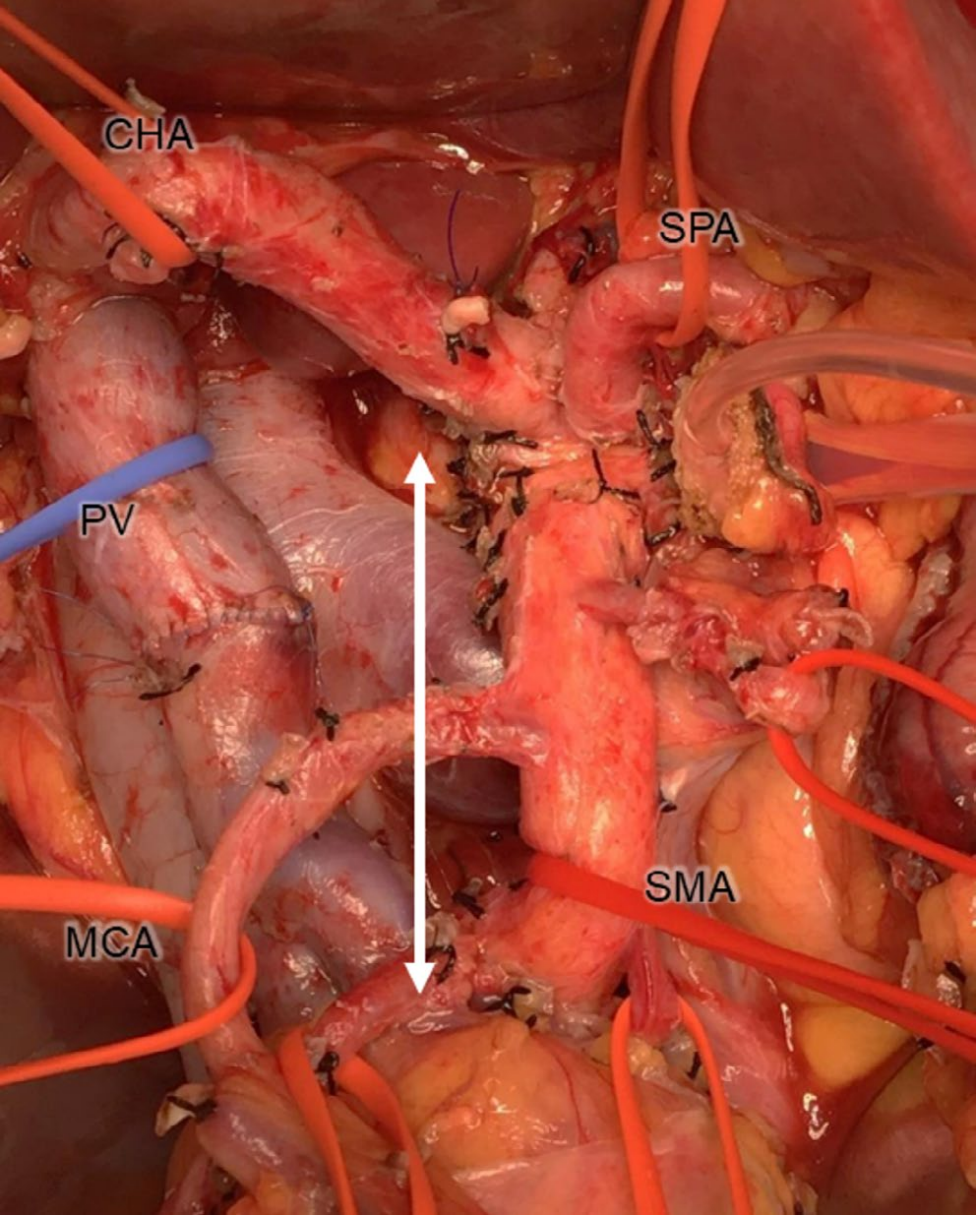
Photography after resection in pancreatoduodenectomy with circumferential lymph node dissection along the SMA (PD‐CLDS). Soft tissue along the SMA between the origin of the SMA and the level of the lower line of the horizontal portion of the duodenum (white arrow) was circumferentially removed. CHA, common hepatic artery; MCA, middle colic artery; PV, portal vein; SMA, superior mesenteric artery; SPA, splenic artery.

A total of 121 patients with PHC underwent pancreatoduodenectomy between 2011 and 2022 at Kanazawa University Hospital. We performed PD‐CLDS between the origin of the SMA and the level of the lower line of the horizontal portion of the duodenum (Figure 2). Eighteen patients who did not undergo circumferential LND along the SMA were excluded. Among these 18 patients, 12 had advanced age or poor performance status, three had a prior history of gastrectomy with LND for gastric cancer, and three underwent insufficient LND because of an association with pancreatitis. The remaining 103 patients were included in the second study.

#### 2.2.2 Pathological Examination

**FIGURE 3:**
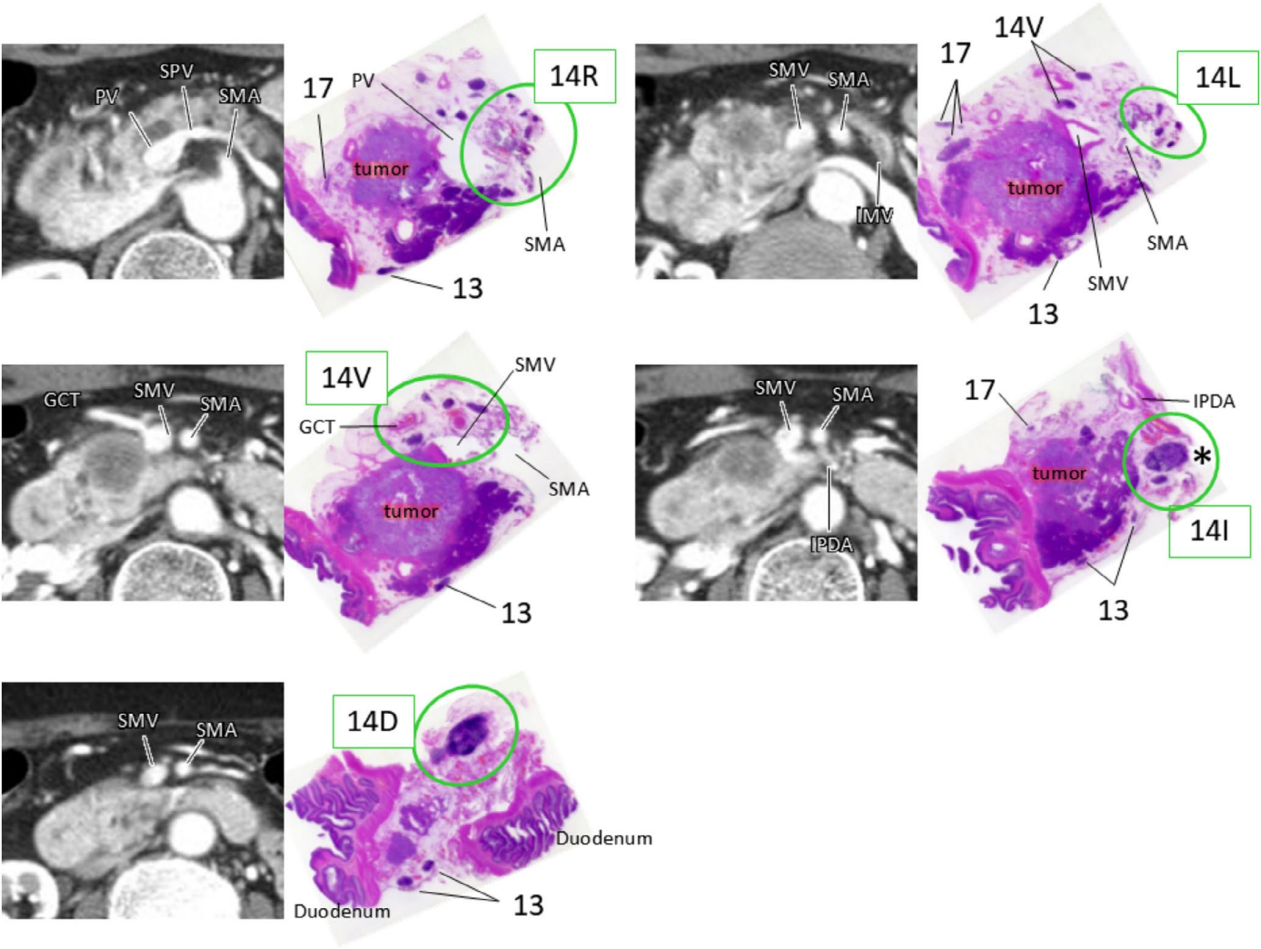
Pathological examination in study 2. Dissected lymph nodes were fixated by formalin without separation from the pancreatic head. Each lymph node was numbered based on its relationship to the resected organs while cross‐referring with corresponding CT axial images (H&E, original magnification x1). Asterisk (*) indicates metastatic lymph node. GCT, gastro‐colic trunk; IMV, inferior mesenteric vein; IPDA, inferior pancreatoduodenal artery; PV, portal vein; PV, portal vein; SMA, superior mesenteric artery; SMV, superior mesenteric vein; SPV, splenic vein.

Pathological examinations were performed in the same manner as in study 1. Dissected LNs were fixated by formalin without separation from the pancreatic head. Each LN was numbered based on its relationship with the resected organs while cross‐referring to the corresponding CT axial images (Figure 3). Dissected LNs were classified into LN station numbers in accordance with *the Classification of Pancreatic Carcinoma (fourth English Edition)* published by the *Japan Pancreas Society* [12]. The main LNs station numbers around the pancreatic head are #8 indicating LNs along the common hepatic artery, #12 indicating LNs in the hepatoduodenal ligament, #13 indicating LNs on the posterior aspect of the head of the pancreas, #14 indicating LNs along the SMA, and #17 indicating LNs on the anterior surface of the head of the pancreas.

The LNs along the SMA were also subclassified into five lymphatic basins according to our original classification defined in the first study. We investigated the incidence of metastasis to each LN station and lymphatic basin along the SMA.

#### 2.2.3 Efficacy Index of Dissected LNs

To evaluate the efficacy of dissection at each LN station or lymphatic basin, we estimated the efficacy index in accordance with the method proposed by Sasako [13]. The efficacy index of each LN station or lymphatic basin was calculated by multiplying the incidence of metastasis to the station or basin with the 5‐year overall survival (OS) rate of patients with metastasis to the station or basin. The OS was calculated as the period from the date of surgery to the date of death or the date of the last observation.

#### 2.2.4 Statistical Analysis

Survival analysis was performed using the Kaplan–Meier method and differences in OS were examined using the log‐rank test. These analyses were performed using SPSS version 19.0 (IBM Corp, Armonk, NY), and *p* < 0.05 was considered statistically significant.

All patients provided written informed consent for inclusion in both studies. These studies were performed in accordance with the tenets of the Declaration of Helsinki. We obtained approval from the Ethics Committee of Kanazawa University Hospital (approval number 2019‐192).

## 3 Results

### 3.1 Study 1: Pathological Study of LNs Along the SMA in PD‐SMAR

#### 3.1.1 Patients' Characteristics

Of the 22 patients who underwent PD‐SMAR (median age, 60 years; range 38–78 years), 14 were male and 8 were female. In accordance with the resectability status based on the National Comprehensive Cancer Network (NCCN) guideline version 2, 2025 [14], one patient was preoperatively classified as resectable (R), 14 as borderline resectable (BR), and 7 as locally advanced unresectable (UR‐LA). Six patients received neoadjuvant therapy (NAT). Five of these 6 patients received chemotherapy, and one received chemoradiotherapy. The numbers of patients in each stage based on the pathological diagnosis were 2 of stage IA, 2 of Stage IB, 2 of stage IIB, 13 of stage III, and 3 of stage IV (all 3 patients had distant LN metastasis), according to the Union for International Cancer Control (UICC)‐TNM Classification 8th edition [15].

#### 3.1.2 Classification of LNs Around the SMA

At the level of the origin of the SMA, several lymph nodes were observed at the anterior‐right side of the SMA. The LNs in this area often existed along the head branch of the dorsal pancreatic artery [16]. We defined this lymphatic basin as number 14R (Figure S1).

At the level of the proximal portion of the SMA, several lymph nodes were observed at the anterior‐left side of the SMA. The LNs in this area often existed along the inferior mesenteric vein. We defined this basin as number 14L (Figure S2).

At the level of the lower border of the pancreatic neck and in front of the superior mesenteric vein (SMV), several LNs were observed at the anterior side of the SMV and arranged to the anterior side of the SMA. The LNs in this area often existed along the gastro‐colic trunk. We defined this basin as number 14V (Figure S3).

At the level of the lower part of the uncinate process of the pancreas, several LNs were observed at the posterior‐left side of the SMA along the inferior pancreatoduodenal artery. We defined this basin as number 14I (Figure S4).

At the level just below the lower edge of the pancreatic head, several LNs were observed in the space between the SMV/SMA and the horizontal portion of the duodenum. The LNs in this area often existed along the first branch of the jejunal vein running behind the SMA (proximal dorsal jejunal vein advocated by Nagakawa et al. [11, 17]). We defined this basin as number 14D (Figure S5).

**FIGURE 4:**
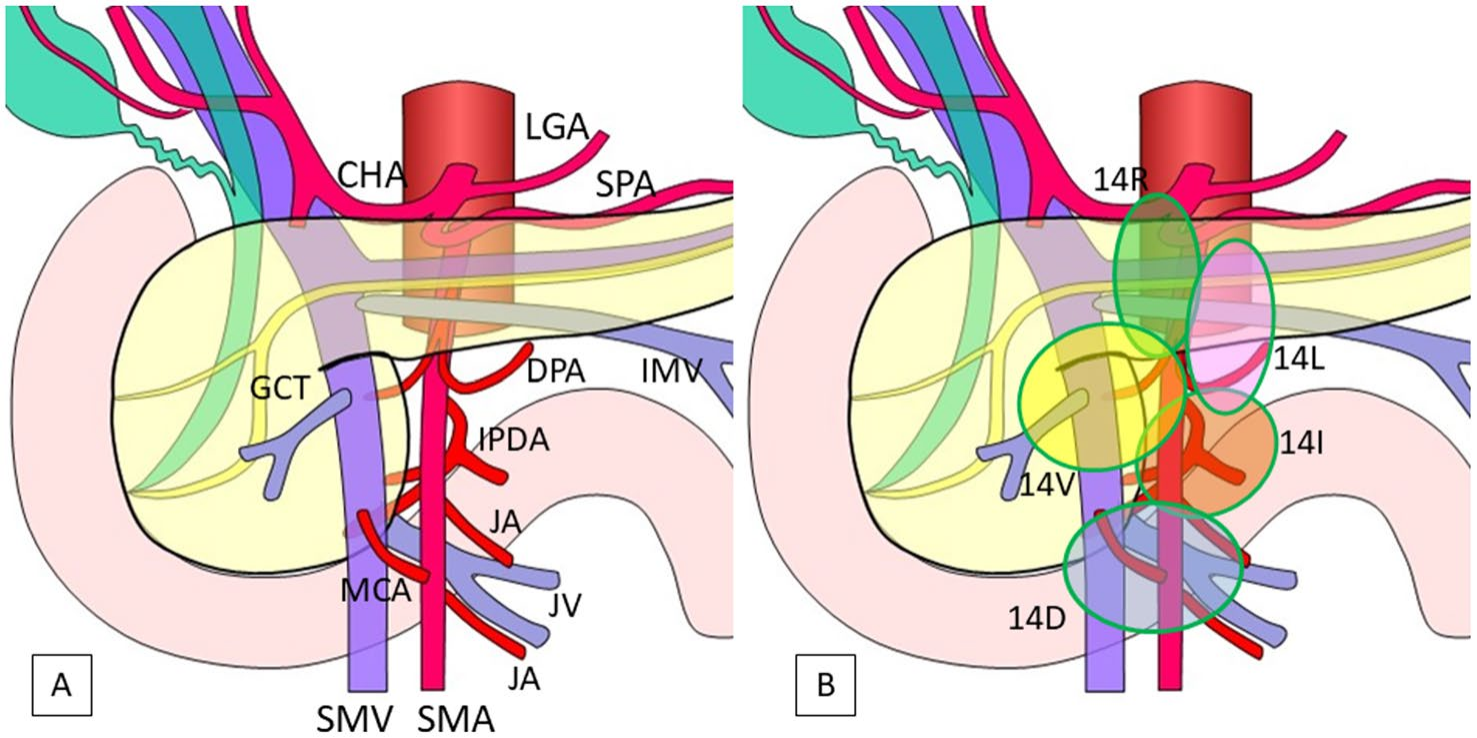
Schema of the comprehensive location of lymphatic basins along the SMA. Lymph nodes along the SMA can be classified into 5 lymphatic basins based on the relationship to the SMA. CHA, common hepatic artery; DPA, dorsal pancreatic artery; GCT, gastro‐colic trunk; IMV, inferior mesenteric vein; IPDA, inferior pancreatoduodenal artery; JA, jejunal artery; JV, jejunal vein; LGA, left gastric artery; MCA, middle colic artery; SMA, superior mesenteric artery; SMV, superior mesenteric vein; SPA, splenic artery.

A schema of the comprehensive locations of lymphatic basins around the SMA is shown in Figure 4 and their landmark structures are summarized in Table 1.

**TABLE 1:** Landmark structures of lymphatic basins around the SMA and incidence of metastasis in PD‐SMAR.

Lymphatic basin	Landmark structure	Incidence of metastasis
14R	Dorsal pancreatic artery	6 (27%)
14L	Inferior mesenteric vein	5 (23%)
14V	Gastro‐colic trunk	7 (32%)
14I	Inferior pancreatoduodenal artery	10 (45%)
14D	Proximal dorsal jejunal vein	7 (32%)

#### 3.1.3 Incidence of LN Metastasis Along the SMA

Any LN metastasis was observed in 16 (73%) of the 22 patients, and any LN metastasis along the SMA was observed in 14 (64%) patients. The numbers of metastatic LNs along the SMA were no node in 8 patients, one node in 5 patients, two nodes in 2 patients, and more than four nodes in 7 patients. Incidence of LN metastasis along the SMA according to the classification for lymphatic basins described above is shown in Table 1. Each of five basins had a metastasis incidence of at least 20% among the 22 patients. Metastasis to 14I was observed in 10 patients (45%), and it was the most frequent basin of metastasis.

### 3.2 Study 2: Evaluation of the Significance of LND Along the SMA

#### 3.2.1 Patients' Characteristics

Of the 103 patients who underwent PD‐CLDS (median age, 68 years; range, 35–80 years), 51 were male and 52 were female. On the basis of the resectability status proposed by the NCCN guideline [14], 52 patients were preoperatively classified as R, 48 as BR, 2 as UR‐LA, and 1 patient was unresectable with distant metastasis (UR‐M). Ninety‐seven patients (94%) underwent NAT. One of the 97 patients underwent preoperative chemoradiotherapy, and the remaining 96 patients underwent preoperative chemotherapy. Both of the 2 UR‐LA patients had partial response with systemic chemotherapy for more than 6 months, and we performed conversion surgery (CS) because R0 resection was expected. One UR‐M patient initially had limited peritoneal metastasis around the pancreatic head which disappeared with systemic chemotherapy for about 1 year, and we performed CS because R0 resection was expected. All 3 patients who underwent CS achieved R0 resection.

#### 3.2.2 Surgical Result

Of the 103 patients, 88 patients (85%) underwent R0 resection. The median operation time was 704 (range 411–995) minutes, intraoperative blood loss was 390 (range 0–1610) mL, and 18 patients (17%) underwent intraoperative blood transfusion. The number of patients in each stage by pathological diagnosis was 7 in stage 0 (pathological complete response), 18 in stage IA, 18 in Stage IB, 4 in stage IIA, 36 in stage IIB, 20 in stage III according to the UICC‐TNM Classification [15].

#### 3.2.3 Incidence of LN Metastasis and Its Prognostic Impact

**FIGURE 5:**
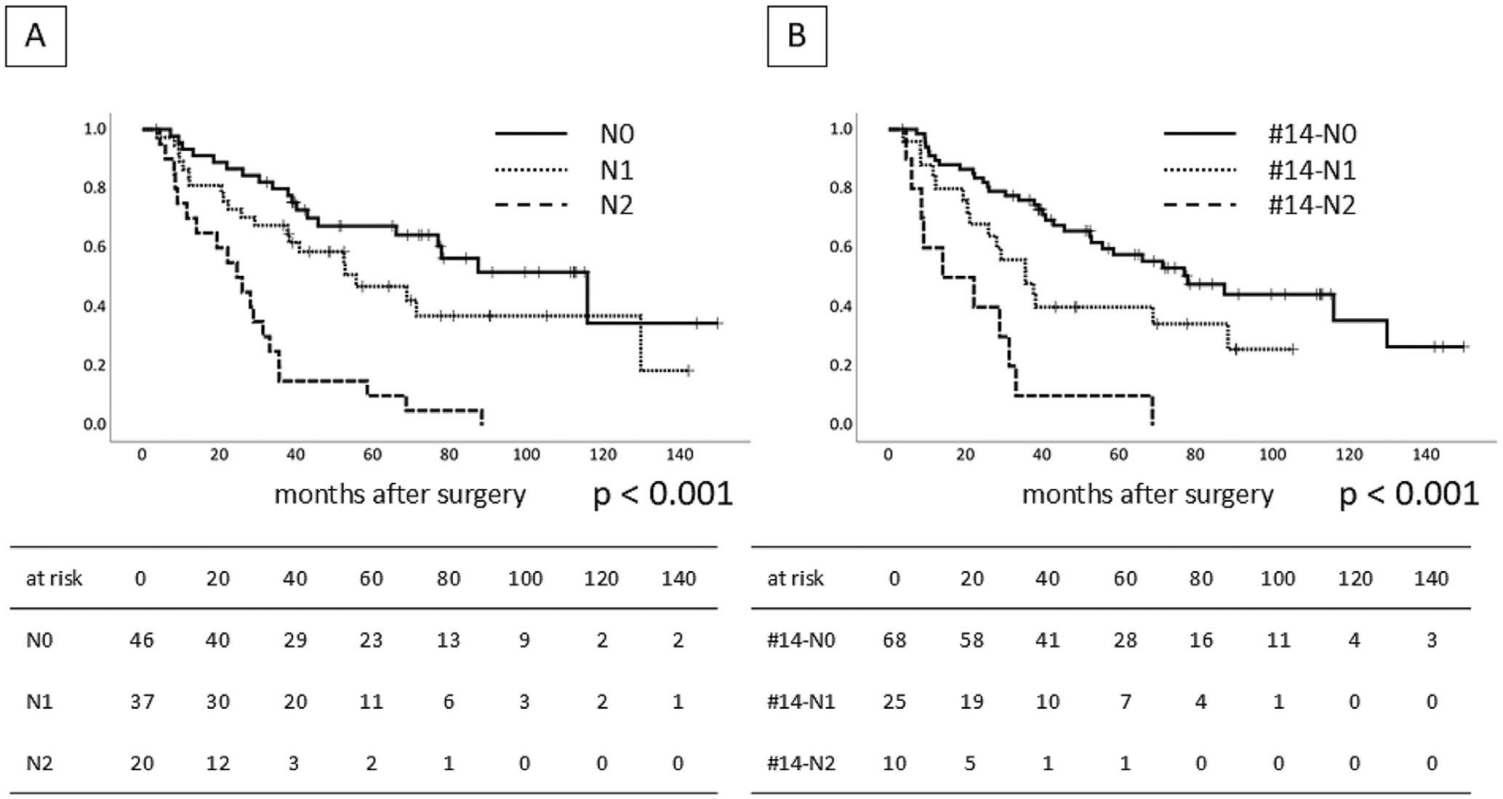
Overall survival after surgery according to the lymph node metastasis. (A) As the disease progressed according to the number of lymph node metastases, the overall survival significantly worsened. (B) The same result was observed regarding the lymph node metastasis at the lymph nodes along the SMA.

Of the 103 patients, 57 patients (55%) had LN metastasis. According to the UICC‐TNM Classification [15], 46 patients were N0, 37 were N1 (metastasis in 1 to 3 nodes), and 20 were N2 (metastasis in more than 4 nodes). Thirty‐five patients (34%) had metastasis at the LN station number 14 (#14), and 25 patients had metastasis in 1 to 3 nodes of #14 (#14‐N1), and 10 patients had metastasis in more than 4 nodes of #14 (#14‐N2). The results of OS after surgery according to the LN metastasis of any dissected LN (Figure 5A) and of #14 (Figure 5B) are shown. The median follow‐up period was 40 (range 4–150) months. The median survival time (MST) and 5‐year OS after surgery for N0, N1, N2 were 115.9, 55.6, 24.6 months and 67.4%, 47.0%, 10.0% respectively. The MST and 5‐year OS of #14‐N0, #14‐N1, #14‐N2 were 77.8, 35.6, 14.0 months and 57.7%, 40.0%, 10.0% respectively.

#### 3.2.4 Efficacy Index of Each LN Station

The incidence of LN metastasis in each station and lymphatic basin along the SMA and its efficacy index are shown in Table 2. Station #14 had the highest frequency of metastasis and was identified as the LN station with the highest efficacy index. The incidence and efficacy indices for #13 and #14 were almost the same and the highest among all LN stations. In contrast, station #12 had the lowest incidence of metastasis and efficacy index.

**TABLE 2:** Incidence of metastasis to each lymph node station and lymphatic basin along the SMA and its efficacy index in PD‐CLDS.

Lymph node station/lymphatic basin	Incidence of metastasis (%)	5‐year survival, with metastasis (%)	Efficacy index
#8	11	27.3	3.003
#12	5	0	0
#13	32	32.3	10.336
#14	34	31.4	10.676
14R	13	23.1	3.003
14L	2	0	0
14V	10	40.0	4.000
14I	19	25.0	4.750
14D	14	14.3	2.002
#17	17	28.2	4.794

*Note:* #8, LNs along the common hepatic artery; #12, LNs in the hepatoduodenal ligament; #13, LNs on the posterior aspect of the head of the pancreas; #14, LNs along the SMA; #17, LNs on the anterior surface of the head of the pancreas.

Of the five lymphatic basins along the SMA, 14I had the highest frequency of metastasis and was the lymphatic basin with the highest efficacy index. In contrast, 14L showed the lowest value of both incidence of metastasis and efficacy index.

## 4 Discussion

Radical surgery for gastrointestinal cancer requires resection with a certain margin for direct cancer invasion, which is recognized as R0 resection [18, 19], and systematic LND of the regional LNs [1, 2, 3, 4]. Although systematic prophylactic LND has been established for other gastrointestinal cancers, such as complete meso‐colic excision (CME) for colon cancer [20], total meso‐rectal excision (TME) for rectal cancer [21], and D2 dissection for gastric cancer [22], the optimal extent and method of dissection for pancreatic cancer remain unclear [23, 24, 25, 26, 27]. Especially in PHC, extension in the direction of the SMA is considered important, and dissection techniques along the SMA have long been discussed [8, 9, 10, 11]. However, consensus regarding the significance of LND along the SMA is currently lacking.

The detailed anatomical structure of the lymphatic system along the SMA was first reported by Deki H and Sato T in 1988 [28]. Our classification, which has been defined in the first study, is consistent with the knowledge from the anatomical study by Deki H. Our study identified 5 distinct lymphatic basins along the SMA (14R, 14L, 14V, 14I, and 14D) and demonstrated that all of them could harbor metastases in patients with PHC who underwent PD‐SMAR (each with more than 20% incidence). However, the first study included a certain number of patients who had previously undergone surgical resection, of which many had so advanced stages of the disease that the frequency of LN metastasis in this population may not accurately reflect the status of current surgical candidates for pancreatic cancer.

Since we recognized frequent metastasis to the LNs all around the SMA in the first study, we performed PD‐CLDS for standard LND along the SMA for PHC. To accurately evaluate the efficacy index, the target LN stations or lymphatic basins should be dissected in as uniform a manner as possible. We consistently performed PD‐CLDS between the origin of the SMA and the level of the lower line of the horizontal portion of the duodenum using a standardized technique by which we could remove all five lymphatic basins along the SMA for the patients in the second study (Figure 2). Precisely classifying each LN into a certain LN station or lymphatic basin is also essential to accurately evaluate the efficacy index. For accurate classification in pathological analysis, the dissected LNs were fixated by formalin without separation from the pancreatic head. Subsequently, they were numbered based on their relationship with the resected organs, while cross‐referring to the corresponding axial CT images (Figures 1 and 3).

Based on the efficacy index, we found that #14 was the most valuable station for LND in PHC (Table 2). This result means that #14 is the primary LN station, as well as #13 and #17 in PHC. Concerning the lymphatic basins in #14, 14I and 14V showed high efficacy index values, followed by 14R and 14D. However, the efficacy index value of 14L was 0. In both the PD‐SMAR and PD‐CLDS cohorts, all patients with LN metastasis in 14L also had LN metastasis in another basin of #14 (data not shown). Thus, 14L may not be a primary lymphatic basin but a secondary basin, and metastasis in 14L may indicate that the lesion has already progressed to a systemic disease in patients with PHC.

The result of OS in relation to LN metastasis indicates that LN metastasis had a significant impact on the survival of patients with PHC. Similar results were observed regardless of whether all dissected LNs were examined or only LN station #14 was examined (Figure 5). Because the prognosis of #14‐N1 was relatively favorable (5 year‐OS 40%), LND along the SMA was considered useful, particularly in patients with limited cancer spread toward the SMA.

Two studies regarding the efficacy of LND in pancreatic cancer have been published by Japanese institutions [29, 30]. In these studies, the incidence of metastasis in #14 was 26.75% [29] and 24.4% [30], and the efficacy indices of #14 were 2.65 [29] and 1.54 [30]. These values were much lower than our data (incidence 34%, efficacy index 10.676). These differences may be attributable to variations in procedure and extent of LND along the SMA, as well as differences in the methods employed in pathological examinations for classifying LN stations. The survival results in our study were better than those of the two previous studies. We believe that the primary reason for this improvement is that our study included recent patients, and most of them underwent NAT. The uniform application of PD‐CLDS, which allows thorough LND along the SMA for all patients, may also have contributed to the improved prognosis.

Although the results of our study may provide important insights regarding surgical treatment of PHC, there were some limitations. One limitation is that this study was conducted at a single institution with a limited number of patients. An evaluation of the LN metastasis status in patients with PHC undergoing LND based on our concept in a larger cohort would likely have yielded more detailed information regarding the efficacy of LND along the SMA. A clinical trial evaluating appropriate LND for pancreatic cancer is currently underway in Japan [31]. In this clinical trial, the latest Japanese classification [32] is adopted for classifying LN stations. LNs along the SMA are simply divided into 14t and 14op according to the relationship with the primary tumor. This classification was defined from a different perspective than the classification of lymphatic basins defined in this study, so it may be worthwhile to verify the clinical significance of both classifications. Another important limitation is that this study did not directly evaluate the impact of LND itself for PHC but only clarified the relative efficacy of LND between each LN station or lymphatic basin. We may need a randomized clinical trial comparing the survival of the patients with PHC with or without LND around the SMA to prove the genuine impact of LND along the SMA.

In conclusion, LNs along the SMA are a frequent site of metastasis and LND along the SMA demonstrates high efficacy among the regional LNs in PHC.

## Funding

The authors have nothing to report.

## Conflicts of Interest

The authors declare no conflicts of interest.

## Supporting information


**Figure S1:** Definition of 14R. (A) CT image; (B) Photographs of the resected specimen (H&E, original magnification ×1); (C) Schema indicates the level of the resected specimen. At the level of the origin of the SMA, several lymph nodes were observed at the anterior‐right side of the SMA. The lymph nodes of this area often existed along the head branch of the dorsal pancreatic artery. BD, bile duct; CHA, common hepatic artery; DPA, dorsal pancreatic artery; GCT, gastro‐colic trunk; IMV, inferior mesenteric vein; IPDA, inferior pancreatoduodenal artery; JA, jejunal artery; JV, jejunal vein; LGA, left gastric artery; MCA, middle colic artery; SMA, superior mesenteric artery; SMV, superior mesenteric vein; SPA, splenic artery; SPV, splenic vein.

**Figure S2:** Definition of 14L. (A) CT image; (B) Photographs of the resected specimen (H&E, original magnification ×1); (C) Schema indicates the level of the resected specimen. At the level of proximal portion of the SMA, several lymph nodes were observed at the anterior‐left side of the SMA. The lymph nodes of this area often existed along the inferior mesenteric vein. CHA, common hepatic artery; DPA, dorsal pancreatic artery; GCT, gastro‐colic trunk; IMV, inferior mesenteric vein; IPDA, inferior pancreatoduodenal artery; JA, jejunal artery; JV, jejunal vein; LGA, left gastric artery; MCA, middle colic artery; SMA, superior mesenteric artery; SMV, superior mesenteric vein; SPA, splenic artery.

**Figure S3:** Definition of 14V. (A) CT image; (B) Photographs of the resected specimen (H&E, original magnification ×1); (C) Schema indicates the level of the resected specimen. At the level of the lower border of the pancreatic neck and in front of the superior mesenteric vein, several lymph nodes were observed at the anterior side of the SMV and arranged to the anterior side of the SMA. The lymph nodes of this area often existed along the gastro‐colic trunk. CHA, common hepatic artery; DPA, dorsal pancreatic artery; GCT, gastro‐colic trunk; IMV, inferior mesenteric vein; IPDA, inferior pancreatoduodenal artery; JA, jejunal artery; JV, jejunal vein; LGA, left gastric artery; MCA, middle colic artery; SMA, superior mesenteric artery; SMV, superior mesenteric vein; SPA, splenic artery.

**Figure S4:** Definition of 14I. (A) CT image; (B) Photographs of the resected specimen (H&E, original magnification ×1); (C) Schema indicates the level of the resected specimen. At the level of the lower part of the uncinate process of the pancreas, several lymph nodes were observed at the posterior‐left side of the SMA along the inferior pancreatoduodenal artery. CHA, common hepatic artery; DPA, dorsal pancreatic artery; GCT, gastro‐colic trunk; IMV, inferior mesenteric vein; IPDA, inferior pancreatoduodenal artery; JA, jejunal artery; JV, jejunal vein; LGA, left gastric artery; MCA, middle colic artery; SMA, superior mesenteric artery; SMV, superior mesenteric vein; SPA, splenic artery.

**Figure S5:** Definition of 14D. (A) CT image; (B) Photographs of the resected specimen (H&E, original magnification ×1); (C) Schema indicates the level of the resected specimen. At the level just below the lower edge of the pancreatic head, several lymph nodes were observed in the space between the SMV/SMA and the horizontal portion of the duodenum. The lymph nodes of this area often existed along the first branch of jejunal vein running behind the SMA. CHA, common hepatic artery; DPA, dorsal pancreatic artery; DUO, duodenum; GCT, gastro‐colic trunk; IMV, inferior mesenteric vein; IPDA, inferior pancreatoduodenal artery; JA, jejunal artery; JV, jejunal vein; LGA, left gastric artery; MCA, middle colic artery; SMA, superior mesenteric artery; SMV, superior mesenteric vein; SPA, splenic artery.

## Data Availability

The data that support the findings of this study are available from the corresponding author upon reasonable request.
