# Supplementary figures and images for: Status of Lymph Node Metastasis and Efficacy of Lymph Node Dissection Along the Superior Mesenteric Artery in Pancreatic Head Cancer: Clinicopathological Analysis of Patients Undergoing Pancreatoduodenectomy With Circumferential Lymph Nodes Dissection Along the Superior Mesenteric Artery
Source: J Hepatobiliary Pancreat Sci. 2026 Jan 28;33(5):397–405. doi: 10.1002/jhbp.70071 (PMC13206522; doi:10.1002/jhbp.70071)

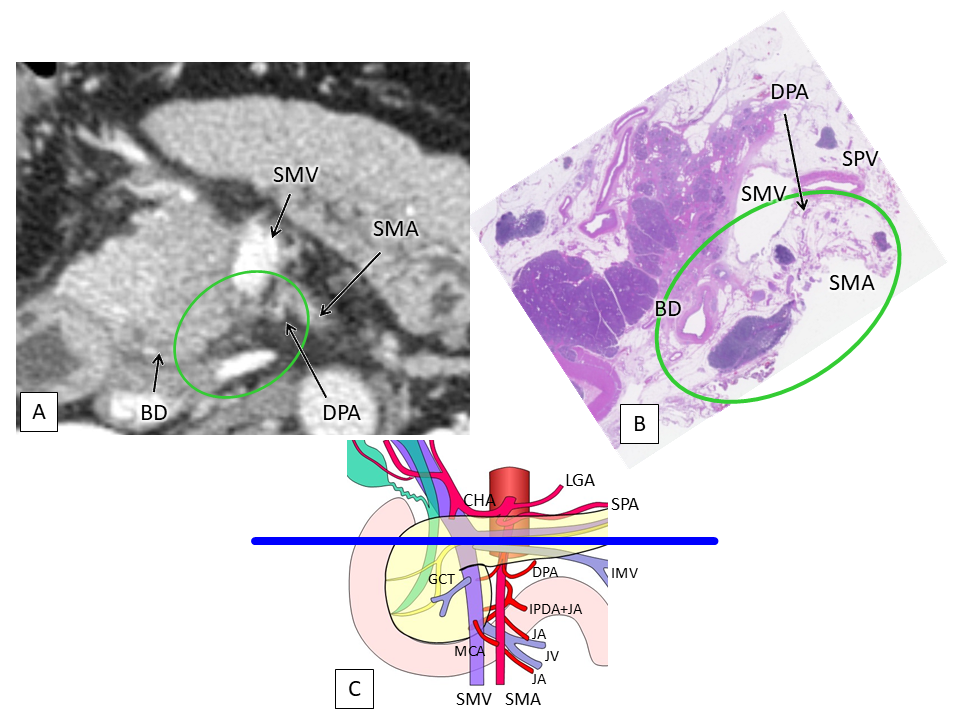

Supplement: Supplementary file 1 — Figure S1: Definition of 14R. (A) CT image; (B) Photographs of the resected specimen (H&E, original magnification ×1); (C) Schema indicates the level of the resected specimen. At the level of the origin of the SMA, several lymph nodes were observed at the anterior‐right side of the SMA. The lymph nodes of this area often existed along the head branch of the dorsal pancreatic artery. BD, bile duct; CHA, common hepatic artery; DPA, dorsal pancreatic artery; GCT, gastro‐colic trunk; IMV, inferior mesenteric vein; IPDA, inferior pancreatoduodenal artery; JA, jejunal artery; JV, jejunal vein; LGA, left gastric artery; MCA, middle colic artery; SMA, superior mesenteric artery; SMV, superior mesenteric vein; SPA, splenic artery; SPV, splenic vein. [file JHBP-33-397-s001.TIF]

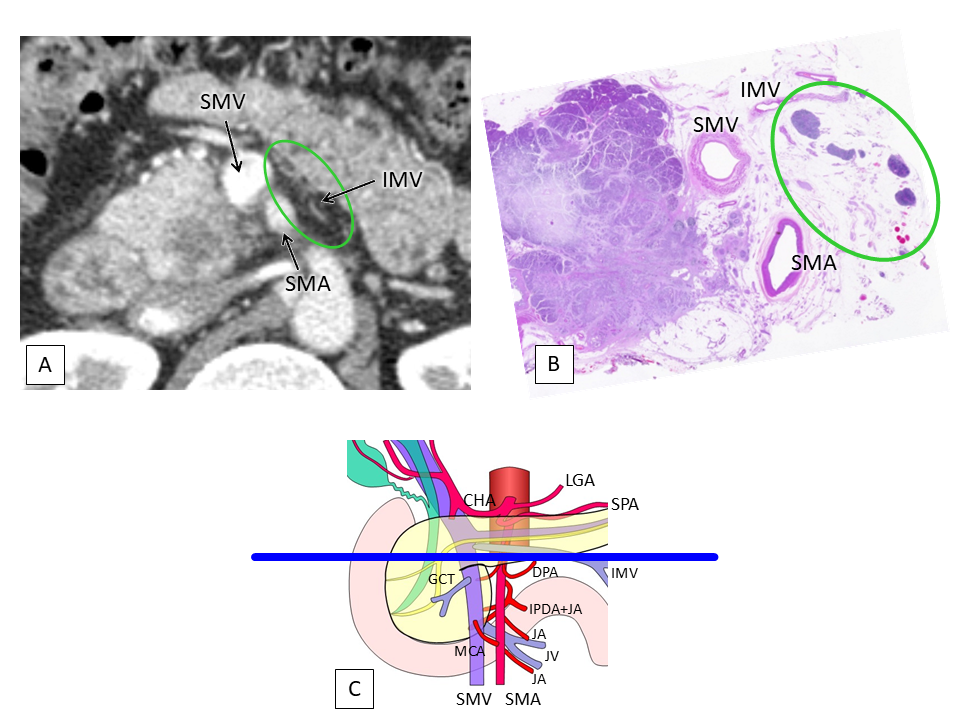

Supplement: Supplementary file 2 — Figure S2: Definition of 14L. (A) CT image; (B) Photographs of the resected specimen (H&E, original magnification ×1); (C) Schema indicates the level of the resected specimen. At the level of proximal portion of the SMA, several lymph nodes were observed at the anterior‐left side of the SMA. The lymph nodes of this area often existed along the inferior mesenteric vein. CHA, common hepatic artery; DPA, dorsal pancreatic artery; GCT, gastro‐colic trunk; IMV, inferior mesenteric vein; IPDA, inferior pancreatoduodenal artery; JA, jejunal artery; JV, jejunal vein; LGA, left gastric artery; MCA, middle colic artery; SMA, superior mesenteric artery; SMV, superior mesenteric vein; SPA, splenic artery. [file JHBP-33-397-s005.TIF]

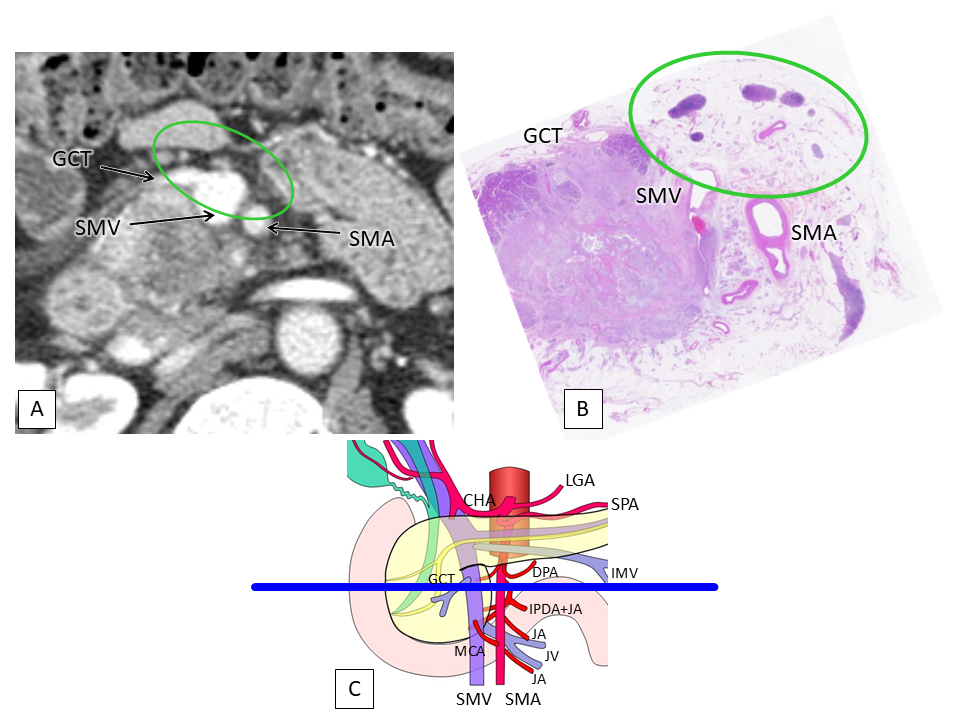

Supplement: Supplementary file 3 — Figure S3: Definition of 14V. (A) CT image; (B) Photographs of the resected specimen (H&E, original magnification ×1); (C) Schema indicates the level of the resected specimen. At the level of the lower border of the pancreatic neck and in front of the superior mesenteric vein, several lymph nodes were observed at the anterior side of the SMV and arranged to the anterior side of the SMA. The lymph nodes of this area often existed along the gastro‐colic trunk. CHA, common hepatic artery; DPA, dorsal pancreatic artery; GCT, gastro‐colic trunk; IMV, inferior mesenteric vein; IPDA, inferior pancreatoduodenal artery; JA, jejunal artery; JV, jejunal vein; LGA, left gastric artery; MCA, middle colic artery; SMA, superior mesenteric artery; SMV, superior mesenteric vein; SPA, splenic artery. [file JHBP-33-397-s003.TIF]

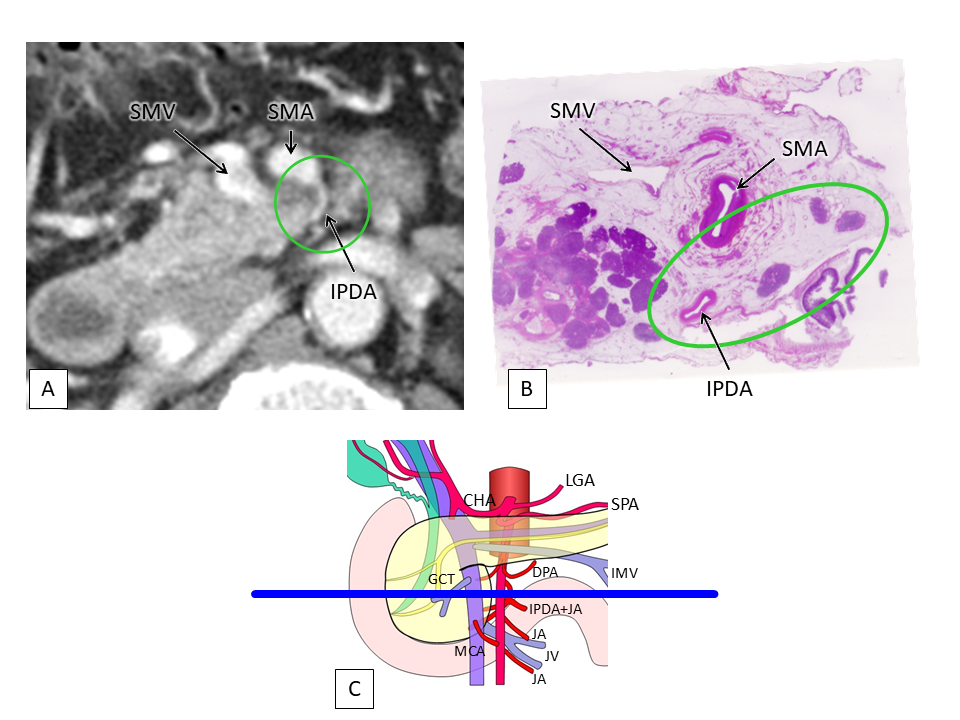

Supplement: Supplementary file 4 — Figure S4: Definition of 14I. (A) CT image; (B) Photographs of the resected specimen (H&E, original magnification ×1); (C) Schema indicates the level of the resected specimen. At the level of the lower part of the uncinate process of the pancreas, several lymph nodes were observed at the posterior‐left side of the SMA along the inferior pancreatoduodenal artery. CHA, common hepatic artery; DPA, dorsal pancreatic artery; GCT, gastro‐colic trunk; IMV, inferior mesenteric vein; IPDA, inferior pancreatoduodenal artery; JA, jejunal artery; JV, jejunal vein; LGA, left gastric artery; MCA, middle colic artery; SMA, superior mesenteric artery; SMV, superior mesenteric vein; SPA, splenic artery. [file JHBP-33-397-s002.TIF]

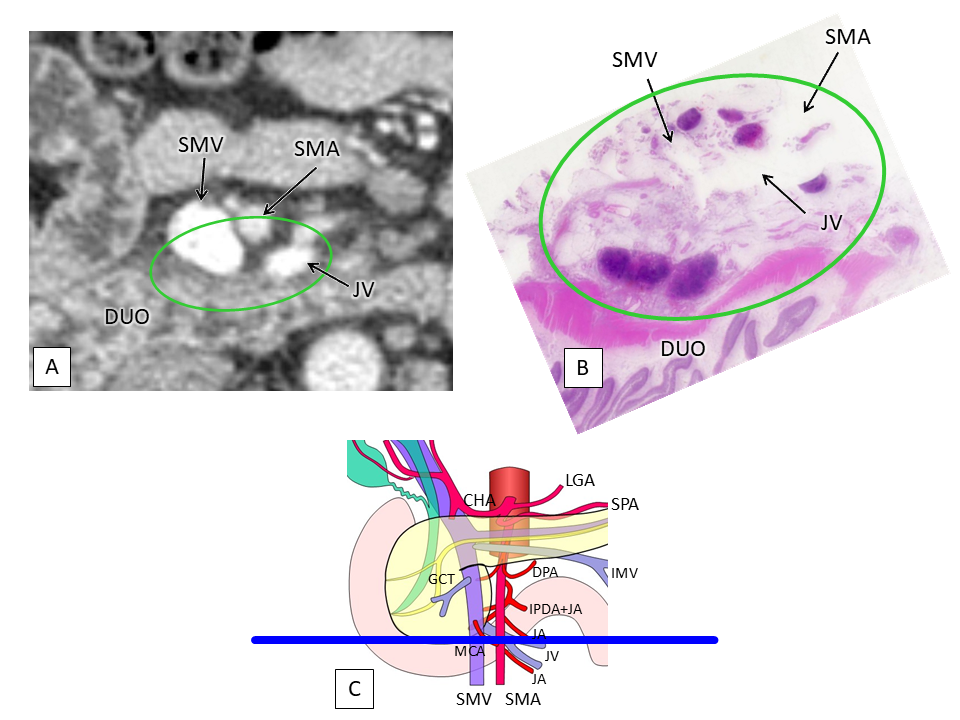

Supplement: Supplementary file 5 — Figure S5: Definition of 14D. (A) CT image; (B) Photographs of the resected specimen (H&E, original magnification ×1); (C) Schema indicates the level of the resected specimen. At the level just below the lower edge of the pancreatic head, several lymph nodes were observed in the space between the SMV/SMA and the horizontal portion of the duodenum. The lymph nodes of this area often existed along the first branch of jejunal vein running behind the SMA. CHA, common hepatic artery; DPA, dorsal pancreatic artery; DUO, duodenum; GCT, gastro‐colic trunk; IMV, inferior mesenteric vein; IPDA, inferior pancreatoduodenal artery; JA, jejunal artery; JV, jejunal vein; LGA, left gastric artery; MCA, middle colic artery; SMA, superior mesenteric artery; SMV, superior mesenteric vein; SPA, splenic artery. [file JHBP-33-397-s004.TIF]
